# Coevolution of host resistance and pathogen exploitation in a propagule-mediated infection model

**DOI:** 10.1371/journal.pcbi.1013999

**Published:** 2026-03-10

**Authors:** Prerna Singh, Justin Sheen, Chadi M. Saad-Roy, Michael Z. Levy, C. Jessica E. Metcalf

**Affiliations:** 1 Evolutionary and Integrative Ecology, Leibniz Institute of Freshwater Ecology and Inland Fisheries, Berlin, Germany; 2 Department of Ecology and Evolutionary Biology, Princeton University, Princeton, New Jersey, United States of America; 3 Department of Global Health and Population, Harvard T.H. Chan School of Public Health, Boston, Massachusetts, United States of America; 4 Miller Institute for Basic Research in Science, University of California, Berkeley, California, United States of America; 5 Department of Integrative Biology, University of California, Berkeley, California, United States of America; 6 Department of Mathematics, University of British Columbia, Vancouver, British Columbia, Canada; 7 Department of Microbiology and Immunology, University of British Columbia, Vancouver, British Columbia, Canada; 8 Biodiversity Research Centre, University of British Columbia, Vancouver, British Columbia, Canada; 9 Department of Biostatistics, Epidemiology and Informatics, University of Pennsylvania, Philadelphia, Pennsylvania, United States of America; University of Auckland, NEW ZEALAND

## Abstract

Host populations often face infection risk from pathogens that can persist in the environment as free-living propagules. We develop a population-level model to understand how host resistance - defined as reduced susceptibility to infection - evolves in response to the exploitation strategy of a pathogen where transmission occurs exclusively via environmental propagules. Using an adaptive dynamics framework, we analyze how the coevolution of host resistance and pathogen exploitation strategy unfolds under the following fitness costs: reduced survival associated with investment in resistance reflected by additional background mortality for the host; and reduced average lifespan represented by increased infected host mortality for the pathogen. Calculating individual host and pathogen invasion fitness expressions using standard invasion analysis, we track how stable levels of investment in host resistance vary across different infection scenarios. We found that costly resistance is disfavoured when pathogen encounters are excessively high, with maximal resistance selected at intermediate levels of transmission. Coevolutionary feedbacks between host resistance and pathogen exploitation can lead to diverse outcomes, including stable evolutionarily singular strategies and, under weakly accelerating costs, evolutionary branching that generates coexistence in the resistance trait. We further quantify how coevolution shapes the equilibrium density of free propagules, revealing conditions under which coevolution suppresses or amplifies pathogen prevalence in comparison to non-evolving scenarios. Overall, our model framework built on survival-based costs offers testable predictions for environmentally transmitted host-pathogen systems.

## Author summary

Hosts generally acquire infection in two primary ways: by coming into contact with their infected counterparts or by encountering free pathogen propagules in the environment. In this study, we developed a population-level model based on a host-pathogen system in which the pathogen can exist both within infected hosts and as free propagules, but transmission occurs exclusively through environmental propagules. We explore how hosts evolve resistance in response to exploitation strategies of the pathogen under the following trade-offs: resistant hosts experience reduced survival, while highly exploitative pathogens shorten the lifespan of their infected hosts. Our analysis reveals that maximal host resistance will evolve at intermediate infection pressures, reflecting a balance between the benefits of avoiding infection and the fitness costs paid as reduced survival. Furthermore, resistance is minimized at conditions reflecting lower population densities, i.e., when mortality rates of susceptible hosts, infected hosts, or free pathogen propagules are heightened. Under certain trade-off shapes, diversification in resistance traits can occur, leading to coexistence of host strains with distinct resistance levels. We also demonstrate the effect of coevolution on the density of free pathogens in the environment, leading to amplified or suppressed levels of infection depending upon varying transmission and mortality parameters. Overall, this work provides a general framework for understanding host-pathogen coevolution in environmentally transmitted systems such as *Daphnia magna-Pasteuria ramosa* and amphibian chytridiomycosis caused by fungal pathogen *Batrachochytrium dendrobatidis*, as well as other diseases involving free-living infectious stages, offering useful eco-evolutionary insights.

## Introduction

Characterizing how host immunity and pathogen virulence coevolve is a classic problem in evolutionary ecology. Theoretical models indicate that the strength of host defense can shape pathogen virulence evolution [1–3], a claim supported empirically. For example, immune defense in rats selects for reduced virulence of a protozoan pathogen [4]; in mice, immune pressure accelerates virulence evolution in a malaria species [5]; and in grapevine, partial resistance selects for more aggressive strains of *Plasmopara viticola* characterized by shorter latency periods and increased spore production [6]. Most population-level models exploring the coevolutionary dynamics of host immunity and pathogen virulence have centered on themes such as genetic specificity and matching dynamics [7,8], spatial patterns of adaptation [9,10], and multi-species interactions [11]. Host-pathogen coevolution has been widely studied in systems where pathogens rely solely on their hosts for transmission [12–15], and some models have specifically examined the coevolution of host resistance and pathogen virulence in such obligate interactions [16,17]. In contrast, models of pathogens with both within-host and free-living stages - where transmission occurs either through direct host contact or environmental propagules - have focused mainly on the evolution of pathogen virulence strategies [18–23], leaving the evolution of host defense in response to pathogen strategies largely unexplored. We aim to bridge this gap by explicitly investigating the coevolution of host resistance and pathogen exploitation strategy in a system where infection occurs exclusively via free-living propagules.

Many pathogens transmit via free-living propagules, including human parasites and bacteria such as *Giardia*, soil-transmitted helminths, *Vibrio cholerae*, and *Bacillus anthracis* [24–27], as well as well-studied systems like *Daphnia-Pasteuria* and plant diseases such as downy mildew and rusts [6,28,29]. In such systems, susceptible hosts can get infected either through direct contact with infected individuals or through exposure to free-living pathogen propagules. The significance of either pathogen transmission route varies across different disease dynamics. For instance, both transmission routes are significant in diseases like cholera [30], whereas infection is more likely to occur through the environmental reservoir in water-borne pathogens that lack efficient direct transmission [31]. Theoretical models predict that the heterogeneous selective pressures experienced by pathogens from environmental reservoirs can result in higher overall virulence compared to those existing solely within their hosts [18,19,21,32–34]. However, the coevolutionary feedbacks that arise between host defense and pathogen exploitation under environmental transmission remain largely unexplored and are expected to differ from those observed in obligate direct-transmission systems, a gap we address through our modelling framework.

While most models of host-pathogen evolution impose costs on fecundity [16,35–42], costs acting on mortality are far less frequently incorporated [43–46], despite the empirical evidence rooted mainly in immune-mediated damage and the constitutive costs of host defense [47–50]. Immune-mediate damage is generally caused by the misdirected or excessively activated immune responses, and has been well-investigated as a key cost shaping the evolution of host immunity [44,48,51–54]. By contrast, mechanisms of constitutive immune defense (resistance) that would allow hosts to avoid becoming infected altogether could incur a cost by diverting limited resources from other fitness-related traits [55,56]. In our model, increased host resistance - defined as reduced susceptibility to infection, incurs fitness cost in terms of additional background mortality of susceptible hosts, capturing a constitutive cost of defense and is consistent with the assumptions used in [46] and [45]. This resistance-survival trade-off reflects the energetic and physiological burdens of maintaining or deploying immune defenses, which can compromise host survival even in the absence of infection. In parallel, the pathogen experiences its own trade-off. For pathogens that exist both within hosts and in free-living stages, replication rates are constrained by a trade-off between propagule production and virulence - higher exploitation increases the release of infectious stages but shortens host survival [18,57,58]. This classical transmission-virulence (or propagule production-host survival) trade-off has been extensively explored in theoretical models and has also been observed empirically. For instance, [59] showed that bacterial virulence decreases under relaxed selection in environmental reservoirs, while [22] developed a theoretical model demonstrating how pathogen growth in reservoir environments feeds back on the evolution of virulence. Similar exploitation-based trade-offs have been captured in models of HIV evolution [60–62], and in superinfection models involving competition for host target cells [63]. Building on this, we model a pathogen exploitation strategy that promotes propagule production at the cost of heightened mortality of infected hosts, i.e., a reduced pathogen lifespan within hosts. Our approach accounts for both pathogen-driven resource competition [64], and the costs imposed by immune-mediated damage from the host perspective [65], allowing us to explore how these interacting costs shape the coevolution of host resistance and pathogen exploitation.

Our framework is broadly applicable to host-pathogen systems in which infection occurs via free-living propagules rather than direct host-to-host contact. By integrating an adaptive dynamics framework [66] into a population dynamics model of hosts and free pathogen propagules, we analyze how trade-offs associated with immune-mediated damage and pathogen-induced death jointly determine the coevolution of host and environmentally transmitted pathogen strategies. Additionally, we examine how factors such as pathogen transmission and intrinsic mortality rates of different populations affect the optimal host investment in resistance. Our framework captures the ecological feedback between immune investment and pathogen prevalence–an essential component of within-host evolutionary dynamics. Finally, we identify conditions that foster polymorphism in resistance as a host defense strategy, leading to the coexistence of host strains with different levels of immune defense.

## Model description and analysis

**Fig 1:**
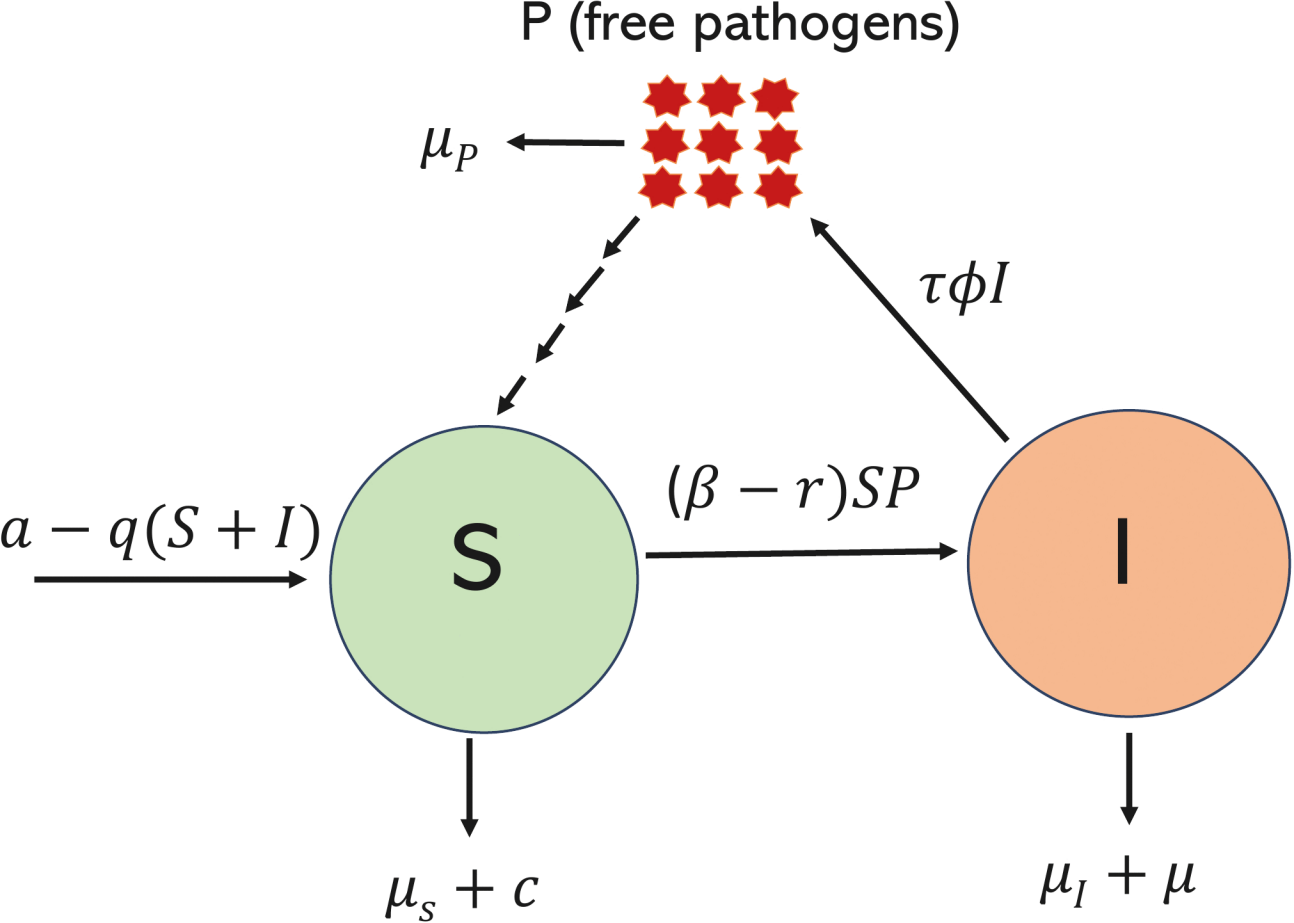
Model schematic.

Building on existing models of pathogen virulence considering both within-host and environmental pathogen stages [21,22], we extend this framework to develop a coevolutionary model of host resistance and pathogen exploitation strategy structured around host immune-mediated damage and pathogen survival. Our model matches closely with the systems such as *Daphnia magna-Pasteuria ramosa*, where transmission can occur solely via environmentally borne spores released from infected hosts [67]. The variables here are population densities of susceptible hosts (*S*), infected hosts (*I*), and free pathogens (*P*). The schematic diagram of the model is shown in Fig 1, and the corresponding system of differential equations describing the population dynamics is given in system 1.


$$\frac{dS}{dt}=(a-q(S+I))S-(\beta -r)SP-\mu_{S}S-c(r)S,$$ (1)

$$\frac{dI}{dt}=(\beta -r)SP-\mu_{I}I-\mu (\phi )I,$$ (2)

$$\frac{dP}{dt}=\tau \phi I-\mu_{P}P.$$ (3)

Here we assume that the reproduction rate *a* of susceptible hosts is density-dependent, meaning that it decreases as the population grows due to competition for limited resources *q*, and these hosts die at background mortality rate $\mu_{S}$. The mass action term βSP reflects the process of infection, where *β* is the rate at which free-living pathogens come in direct contact with susceptible hosts. We model the evolution of host resistance by factor *r* which reduces the effective contact rate between free pathogens and susceptible hosts, thereby reducing the possibility of successful infection. We consider the role of environmental transmission only and ignore the possibility of infection transmission through contact with infected hosts. Infected hosts die naturally at rate $\mu_{I}$ and suffer additional mortality (virulence) due to their resources being invested in the host exploitation strategy *ϕ*. The trait *ϕ* represents the intensity with which the pathogen exploits host resources during infection, such that higher values correspond to faster replication within the host. Each infected host releases free pathogen propagules into the environment at a constant rate τI, where *τ* denotes the baseline production rate per infected host. Furthermore, free pathogens are cleared from the environment at rate $\mu_{P}$.

To explore how resistance as a host defense strategy evolves in response to the pathogen exploitation strategy, we explicitly include trade-offs in both the host and the pathogen population, representing the physiological or ecological costs associated with these strategies. For the host, investment in resistance diverts resources away from other fitness-related processes, reflected by an increment in its background mortality rate c(r). The pathogen population, on the other hand, exploits the host resources to replicate faster within their hosts, which leads to greater propagule shedding via lysis or host breakdown mechanisms [19,68]. While this strategy accelerates the turnover of infected hosts, it incurs a cost via higher infected host mortality μ(ϕ), i.e., a shorter infectious period. A summarised description of the parameters is given in Table 1.

**Table 1:** Description of model parameters and baseline values used in the simulations.

Parameter	Definition	Default value
*a*	susceptible host birth rate	2
*q*	density-dependent crowding effect	0.2
*β*	infection rate of susceptible hosts	3
$\mu_{S}$	natural death rate of susceptible hosts	0.2
$\mu_{I}$	natural death rate of infected hosts	0.2
$\mu_{P}$	free pathogen decay rate	0.2
*τ*	propagule release rate	0.5
*μ*	infection-induced death rate	varies
*ϕ*	pathogen exploitation strategy	varies
*r*	host resistance	varies
*c*	cost of resistance	varies

System 1 has two non-trivial equilibrium solutions. One is the disease-free equilibrium $\left(S^{0},I^{0},P^{0})=(\frac{a-c(r)-\mu_{S}}{q},0,0\right)$ and another is the endemic equilibrium $\left(S^{*},I^{*},P^{*}\right)$, where


$$S^{*}=\frac{-(\mu (\phi )+\mu_{I})\mu_{P}}{(r-\beta )\tau \phi },$$

$$I^{*}=-\frac{\mu_{P}\left(q\left(\mu (\phi )+\mu_{I}\right)\mu_{P}+(r-\beta )\tau \phi (a-c(r)-\mu_{S})\right)}{(r-\beta )\tau \phi \left((r-\beta )\tau \phi -q\mu_{P}\right)},$$

$$P^{*}=\frac{-q\left(\mu (\phi )+\mu_{I}\right)\mu_{P}+(r-\beta )\tau \phi (-a+c(r)+\mu_{S})}{(r-\beta )\left((r-\beta )\tau \phi -q\mu_{P}\right)}.$$

The parameter values were chosen to ensure biologically meaningful outcomes, specifically that


$${ℛ}_{0}=\frac{(\beta -r)\;S^{0}\;\tau \phi }{(\mu (\phi )+\mu_{I})\;\mu_{P}}=\frac{\left(\beta -r)\;\tau \phi \;(a-c(r)-\mu_{S}\right)}{(\mu (\phi )+\mu_{I})\;\mu_{P}\;q}>1,$$

thereby allowing infection persistence and exploration of a biologically plausible range of ecological and evolutionary behaviors. Integrated datasets are rarely available for environmentally transmitted pathogens, particularly for systems combining within-host exploitation with a free-pathogen environmental stage. In the absence of such estimates, we follow standard modeling assumptions used in host-pathogen evolutionary models with free propagules stage [18,21]. We therefore focus on qualitative eco-evolutionary outcomes rather than inferring system-specific parameters across broad ranges of parameters subject to standard biological constraints. Specifically, we need (i) a feasible disease-free equilibrium $S^{0}>0$, (ii) invasion feasibility ${ℛ}_{0}>1$, and positive equilibrium $(S^{*},I^{*},P^{*})>0$. In addition, we verify that the chosen parameters imply biologically reasonable infection and environmental timescales, as detailed in S1 Text, Sect 1.1. Local stability of the endemic equilibrium is determined using the Routh-Hurwitz criteria, based on a combination of analytical and numerical calculations [69,70] (see S1 Text and S2 Data).

### Adaptive dynamics

We use adaptive dynamics (AD) framework [66,71] to study the coevolutionary dynamics of host resistance trait *r* and pathogen exploitation strategy *ϕ*. AD models how small, successive mutations drive the gradual evolution of traits through ecological interactions and selection pressures. Here, we assume that the resident host strain with strategy (r,c(r)) and the resident pathogen strain with strategy (ϕ,μ(ϕ)) are fixed at their respective equilibria. In this environment, mutant host and pathogen strains with strategies $\left(r_{m},c(r_{m})\right)$ and $\left(\phi_{m},\mu (\phi_{m})\right)$, respectively, appear and potentially invade the resident strains. The possibility of invasion by the mutants (or their positive growth rate in the resident environments) is given by their sign of invasion fitness expressions. We first calculate the mutant system dynamics as given below:


$$\frac{dS_{m}}{dt}=(a-q(S^{*}+I^{*}+S_{m}+I_{m}))S_{m}-(\beta -r_{m})S_{m}(P^{*}+P_{m})-\mu_{S}S_{m}-c(r_{m})S_{m},$$ (4)

$$\frac{dI_{m}}{dt}=(\beta -r_{m})S_{m}(P^{*}+P_{m})-\mu_{I}I_{m}-\mu (\phi_{m})I_{m}.$$ (5)

The invasion fitness expression of the mutant host strain is equivalent to the maximum eigenvalue of the Jacobian matrix of mutant system dynamics (see S1 Text and also [72]), and is given by:


$$s(r,r_{m})=a-q(S^{*}(r)+I^{*}(r))-(\beta -r_{m})P^{*}(r)-\mu_{S}-c(r_{m}).$$ (6)

If $s(r,r_{m})>0$, the mutant host strategy can either invade and replace the resident strategy or coexist with it. Otherwise, if $s(r,r_{m})<0$, the mutant fails to establish and eventually goes extinct. Through a series of mutation and invasion events, the host resistance strategy $r^{*}$ evolves until it reaches an evolutionary singular point (a temporary stop point of evolution). Mathematically, these points are derived where the fitness gradient equals zero, i.e., are the solutions to $\frac{\partial s}{\partial r_{m}}{|}_{r=r_{m}=r^{*}}=0$.

For the pathogen fitness expression, we compute the basic reproduction number of the mutant pathogen strain with strategy $\phi_{m}$ which is introduced into the resident environment set at its equilibrium strategy *ϕ*. Using the classical invasion analysis by [73] and [62], we derive the basic reproduction number ${ℛ}_{I}(\phi ,\phi_{m})$ given by,


$${ℛ}_{I}(\phi ,\phi_{m})=\frac{{ℛ}_{0}(\phi_{m})}{{ℛ}_{0}(\phi )}=\frac{\tau \phi_{m}(\beta -r)S^{*}(\phi )}{\mu_{P}(\mu_{I}+\mu (\phi_{m}))}=\frac{\phi_{m}(\mu_{I}+\mu (\phi ))}{\phi (\mu_{I}+\mu (\phi_{m}))},$$ (7)

where $S^{*}(\phi )$ is the resident susceptible host density at endemic equilibrium. Using the next-generation method of [70], we derive the following expression which is sign equivalent to the invasion fitness of the pathogen mutant with strategy $\phi_{m}$ (also see [74]):


$$p(\phi ,\phi_{m})=\frac{\phi_{m}(\mu_{I}+\mu (\phi ))}{\phi (\mu_{I}+\mu (\phi_{m}))}-1={ℛ}_{I}(\phi ,\phi_{m})-1.$$ (8)

The mutant pathogen strain can invade the resident strain with strategy *ϕ* if and only if $p(\phi ,\phi_{m})>0$, i.e., when ${ℛ}_{I}(\phi ,\phi_{m})>1$.

Using small mutations and trait substitutions, coevolutionary dynamics of the host and pathogen traits *r* and *ϕ* over evolutionary time *T* can be approximated via following pair of equations:


$$\frac{dr}{dT}\propto S^{*}\frac{\partial s}{\partial r_{m}}{|}_{r_{m}=r}=\eta_{h}S^{*}\frac{\partial s}{\partial r_{m}}{|}_{r_{m}=r},$$ (9)

$$\frac{d\phi }{dT}\propto I^{*}\frac{\partial p}{\partial \phi_{m}}{|}_{\phi_{m}=\phi }=\eta_{p}I^{*}\frac{\partial p}{\partial \phi_{m}}{|}_{\phi_{m}=\phi },$$ (10)

where $\eta_{h}$ and $\eta_{p}$ represent the rates of mutation of the host and pathogen, respectively. For analytical simplicity and keeping in lines with previous studies [15,75], we consider that both host and pathogen have the same rate of mutations, i.e., $\eta_{h}=\eta_{p}=1$. Both populations coevolve along their respective fitness gradients, until a co-evolutionary singular point $\left(r^{*},\phi^{*}\right)$ is reached where the two gradients become simultaneously zero [76]. The stability of a co-singular strategy pair is assessed in two ways: evolutionary stability (ES), which determines whether a rare mutant can successfully invade, and convergence stability (CS), which predicts whether nearby strategies evolve toward $\left(r^{*},\phi^{*}\right)$ over time [77]. Using AD stability conditions for coevolving species (see S1 Text and [78]), we classify the cosingular strategies into continuously stable strategies (co-CSS, a strategy which is both ES and CS) or the branching points (a strategy which is CS but not ES). For evolutionary branching, however, the respective species must satisfy the additional condition of mutual invasibility (i.e., $\frac{\partial^{2}s}{\partial r\partial r_{m}}<0$ for the host and $\frac{\partial^{2}\phi }{\partial \phi \partial \phi_{m}}<0$ for the pathogen) [66,78].

### Trade-off functions

**Fig 2:**
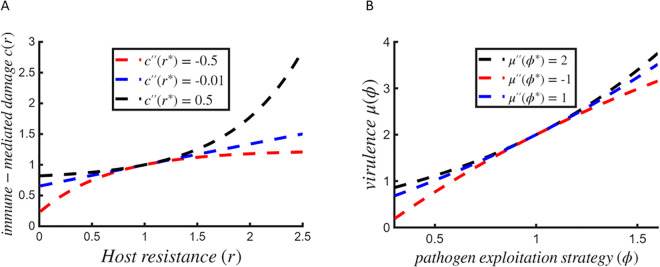
Trade-offs. Plots showing (A) host trade-off function, and (B) pathogen trade-off function, for three different curvature values. Here the slopes are $c^{\prime }(r^{*})=0.3423$ and $\mu^{\prime }(\phi^{*})=2.2$, obtained for the default parameter set and chosen singular strategies $r^{*}=1$ and $\phi^{*}=1$, respectively.

Eco-evolutionary outcomes are rooted in the underlying trade-off structures [79], in our case, for host resistance strategy, *r* and pathogen exploitation strategy, *ϕ* (Fig 2). Given that the shape of trade-offs strongly influences evolutionary outcomes, we use a well-established trade-off function that allows for flexible adjustment of trade-off curvature [72,80]. The cost function reflecting a trade-off between host resistance *r* and mortality rate *c* is given by


$$c(r)=c(r^{*})-\frac{c^{\prime }(r^{*}{)}^{2}}{c^{\prime \prime }(r^{*})}\left(1-e^{\frac{c^{\prime \prime }(r^{*})(r-r^{*})}{c^{\prime }(r^{*})}}\right),$$ (11)

and its shape is illustrated in Fig 2A.

Similarly, for the pathogen, trade-off between its exploitation strategy *ϕ* and infection-induced mortality rate (or virulence) *μ* is given by


$$\mu (\phi )=\mu (\phi^{*})-\frac{\mu^{\prime }(\phi^{*}{)}^{2}}{\mu^{\prime \prime }(\phi^{*})}\left(1-e^{\frac{\mu^{\prime \prime }(\phi^{*})(\phi -\phi^{*})}{\mu^{\prime }(\phi^{*})}}\right),$$ (12)

and its shape is illustrated in Fig 2B. Here, $c^{\prime }(r^{*})$ and $\mu^{\prime }(\phi^{*})$ are the slopes, while $c^{\prime \prime }(r^{*})$ and $\mu^{\prime \prime }(\phi^{*})$ are the curvatures of the underlying trade-offs. The points $\left(r^{*},c(r^{*})\right)$ and $\left(\phi^{*},\mu (\phi^{*})\right)$ correspond to the host and pathogen singular strategies, respectively. We first fix these strategies at chosen values, then determine the slopes such that the fitness gradients vanish at the chosen strategies $r^{*}$ and $\phi^{*}$. Then we adjust the curvatures so that it reflects either accelerating (positive curvature) or decelerating (negative curvature) patterns, which influences the scaling of costs with respect to trait values.

## Results

### Variation in stable investment in host resistance

Initially, we use accelerating trade-offs for both host and pathogen ($c^{\prime \prime }(r^{*})=0.5$, and $\mu^{\prime \prime }(\phi^{*})=0.5$). This choice of curvature values implies that as host resistance or pathogen exploitation increases, the associated fitness costs rise at an increasing rate, making further investment progressively more costly. In consistence with existing evolutionary models [16,39,75,81], our analysis showed that such trade-off shapes yield a continuously stable strategy (CSS), reflecting stable investments in evolving strategies - the primary focus of this study. Furthermore, infection remains at non-zero levels in all cases, indicating endemic persistence across the parameters explored.

**Fig 3:**
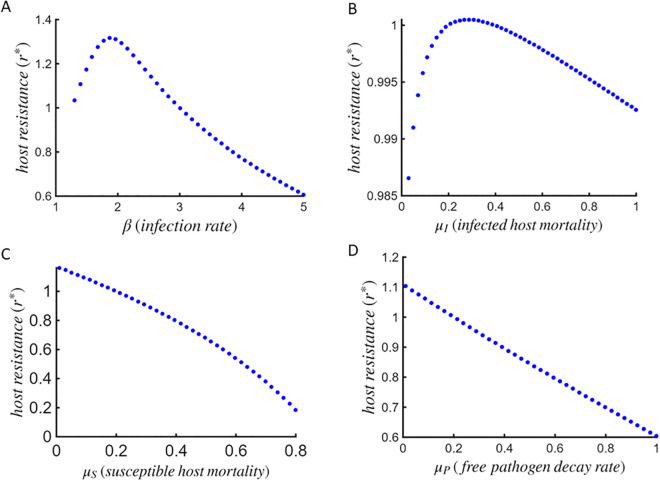
CSS resistance investment patterns. Plots illustrating how CSS investment in resistance varies with (A) the infection rate *β*, (B) the intrinsic mortality rate of infected hosts $\mu_{I}$, (C) susceptible hosts $\mu_{S}$, and (D) free pathogens $\mu_{P}$, when both host and pathogen strategies coevolve. Here, we use $c^{\prime \prime }(r^{*})=0.5$, $\mu^{\prime \prime }(\phi^{*})=0.5$, and remaining parameters are the same as in Table 1.

We begin by investigating how variations in specific life-history traits of the host-pathogen system influence the evolutionarily stable levels of host resistance *r* under coevolution. In particular, we present how increasing rates of host invasion by free pathogens, i.e., infection transmission rate *β*, intrinsic mortality rates of susceptible hosts $\mu_{S}$, infected hosts $\mu_{I}$, and free pathogens $\mu_{P}$ affect the investment in host resistance as CSS strategies (Fig 3).

Results indicate that the host resistance evolves to its maximum value at intermediate infection rates in response to increasing rates of infection by free pathogens (Fig 3A). At low infection rates, the selection advantage of investing in resistance is low, as encounters with free pathogens are limited. However, as infection rate rises, the survival cost incurred by resistant hosts begins to outweigh the benefits of avoiding infection. This creates a negative feedback on further investments in resistance, promoting the strongest selection at intermediate pathogen pressure.

A similar trend is observed with increasing intrinsic infected host mortality rate ($\mu_{I}$): host resistance initially rises sharply with a slight increase in $\mu_{I}$, but subsequently declines, forming an inverted U-shaped pattern (Fig 3B). Initially, an increment in pathogen-induced mortality rates intensify the selection for resistance to prevent host deaths. However, when pathogen virulence is high and infected hosts die more rapidly, the associated cost of mortality would make investments in resistance less advantageous than allowing pathogen spread, thereby diminishing the investments returns in host defense.

On the other hand, increasing mortality rate of susceptible hosts $\mu_{S}$ selects for monotonic decline in the evolutionarily stable level of host resistance (Fig 3C). When background mortality is high, the average lifetime of susceptible hosts is shorter, reducing the fitness benefits of investing in costly resistance. Given the associated costs of mortality, an investment in resistance strategy in such scenarios will further shorten the host lifespan without providing sufficient benefit against infection.

Similarly, an increase in the mortality rate of free pathogens $\mu_{P}$ weakens selection for host resistance (Fig 3D). As free pathogens die more rapidly in the environment, infection risk declines, and the advantage of strong resistance diminishes. Under these conditions, hosts can minimize mortality costs by reducing investment in resistance while still maintaining adequate protection against infection.

We also performed a sensitivity analysis to confirm the robustness of our patterns by computing coevolutionary singular strategies $r^{*}$ across broad ranges of nuisance parameters, retaining the values obtained only at biologically feasible endemic equilibria $(S^{*},I^{*},P^{*})>0$. We found that the qualitative patterns in Fig 3 persist across these varying parameter sets (see S1 Fig).

### Variation in corresponding pathogen abundance

**Fig 4:**
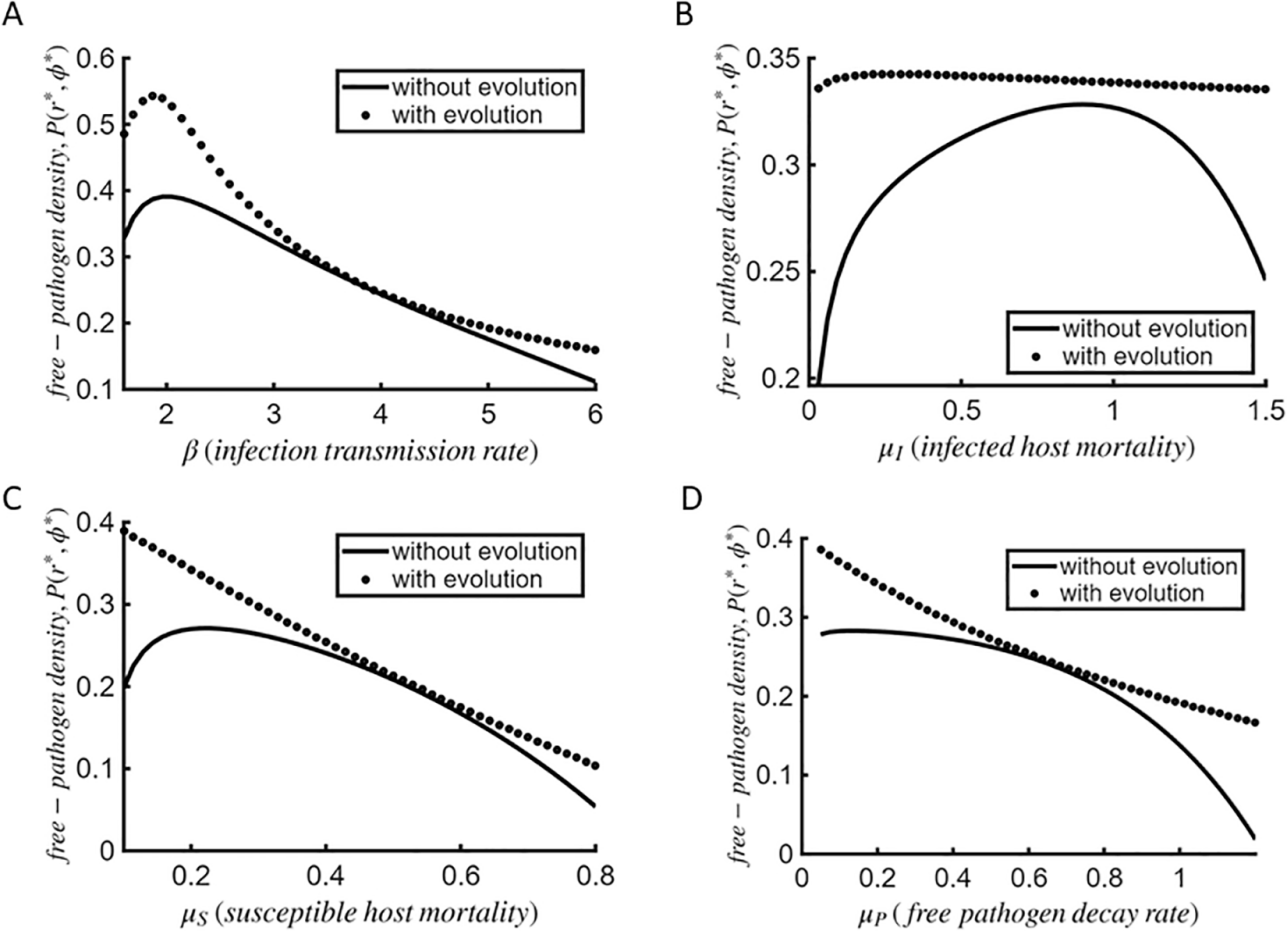
Environmental reservoir pathogen dynamics. Patterns displaying the pathogen abundance in environmental reservoir corresponding to varying (A) infection transmission rate *β*, (B) intrinsic mortality rates of infected hosts $\mu_{I}$, (C) susceptible hosts $\mu_{S}$, and (D) free pathogens $\mu_{P}$. For the “no evolution” case (solid lines), equilibrium pathogen density is calculated at the corresponding parameter set, where *r* and *ϕ* vary within range-space [0.1,1.5]. For “evolution” case (dashed lines), *P* is calculated at the corresponding co-CSS points $\left(r^{*},\phi^{*}\right)$. We use $c^{\prime \prime }(r^{*})=0.5$, $\mu^{\prime \prime }(\phi^{*})=0.5$, and remaining parameters are the same as in Table 1.

The ecological feedbacks between pathogen load and optimal investment in host defense play a crucial role in shaping the coevolutionary trajectory of both the pathogen and its host. As pathogen load fluctuates, selection pressures on host defense strategies adjust accordingly. Conversely, varying levels of investment in resistance mechanisms can alter the infection dynamics, impacting pathogen adaptation and persistence [39]. To explore these links, we analyze the variation in equilibrium density of free pathogen population *P* at corresponding co-CSS points $\left(r^{*},\phi^{*}\right)$ with respect to the parameters governing infection transmission rate *β*, intrinsic mortality rates of infected hosts $\mu_{I}$, susceptible hosts $\mu_{S}$, and free pathogens $\mu_{P}$ (dotted curve, Fig 4). We also compare the equilibrium densities with the scenario when none of the host or pathogen strategies evolve (solid curve, Fig 4). In that case, the parameters representing host resistance and pathogen exploitation strategy are not evolving singular strategies and are simply parameter values varying within a feasible range-space, but bound by their usual costs to survival.

In the case of both evolving and non-evolving strategies, population density of free pathogens initially increases with infection rate *β*, reaches a peak, and then gradually declines (Fig 4A). While higher infection rates facilitate pathogen spread and increase overall abundance initially, excessively high rates of infection spread will lead to increased mortality of infected hosts, ultimately limiting pathogen proliferation and reducing overall pathogen abundance. When both host resistance and pathogen exploitation evolve, the decline in pathogen density is gradual and saturating, whereas in the non-evolving case it is steeper. This distinction arises because evolving host resistance counteracts overexploitation by pathogen, thereby altering pathogen abundance and stabilizing pathogen density at higher infection rates.

For increasing infected host mortality rate, pathogen abundance forms a downward U-shaped curve in the case of non-evolving strategies (Fig 4B). At first, the death of infected hosts leads to greater release of free pathogen propagules through lysis or host breakdown, temporarily boosting pathogen abundance. However, with further increments in mortality, the rapid loss of infected hosts reduces opportunities available for pathogen replication, ultimately leading to a decline in their abundance. In contrast, when both host and pathogen strategies are allowed to evolve, pathogen density exhibits a shallow initial dip followed by a marginal and steady decline. This pattern reflects evolutionary feedbacks adjusting both host resistance and pathogen exploitation in response to rising mortality. Hosts reduce investment in costly resistance as high mortality lessens its benefits (see Fig 3B), while pathogens adopt less aggressive strategies to prolong infection duration–together stabilizing pathogen density.

Next, free-pathogen density pattern for the “no-evolution” scenario shows a small initial increase followed by a continuous decline with increasing susceptible host mortality rate $\mu_{S}$ (Fig 4C). At low levels of $\mu_{S}$, a small decline in susceptible hosts raises the proportion of infected hosts, leading to a brief incline in pathogen density. However, further depletion of susceptible hosts restrict the opportunities for new infections, causing a steady decline in the free pathogen abundance. In contrast, when both strategies evolve, pathogen density declines more rapidly with increasing susceptible host mortality rate. As hosts reduce investment in resistance with increasing $\mu_{S}$ (see Fig 3C), the resistance-mortality trade-off balances out the background mortality rate, eliminating the initial pathogen density rise as seen in the non-evolving case. As pathogen replication opportunities shrink with increasing death of susceptible hosts, free-pathogen density declines continuously. This outcome emphasizes that reduced opportunities for pathogen replication have a much stronger impact on pathogen abundance than variation in host resistance.

Finally, an increase in the mortality rate of free pathogens leads to a monotonic decline in pathogen abundance in both evolving and non-evolving strategy scenarios, as intuitively expected (Fig 4D). While the decline is sharper when no strategies evolve, driven by direct loss of pathogenic particles–it is more gradual when evolution is allowed. Evolutionary feedbacks mitigate the decline, as hosts reduce resistance and pathogens adjust exploitation strategies to sustain infection under increasing mortality rate of free pathogens.

### Coexistence of polymorphic host strains

From our analytical analysis, we observed that required conditions for evolutionary branching holds for the susceptible host but not for the infected host population (see S1 Data). In particular, for a limited range of trade-off shapes, the host strains with distinct resistance levels can coexist in the population via branching.

**Fig 5:**
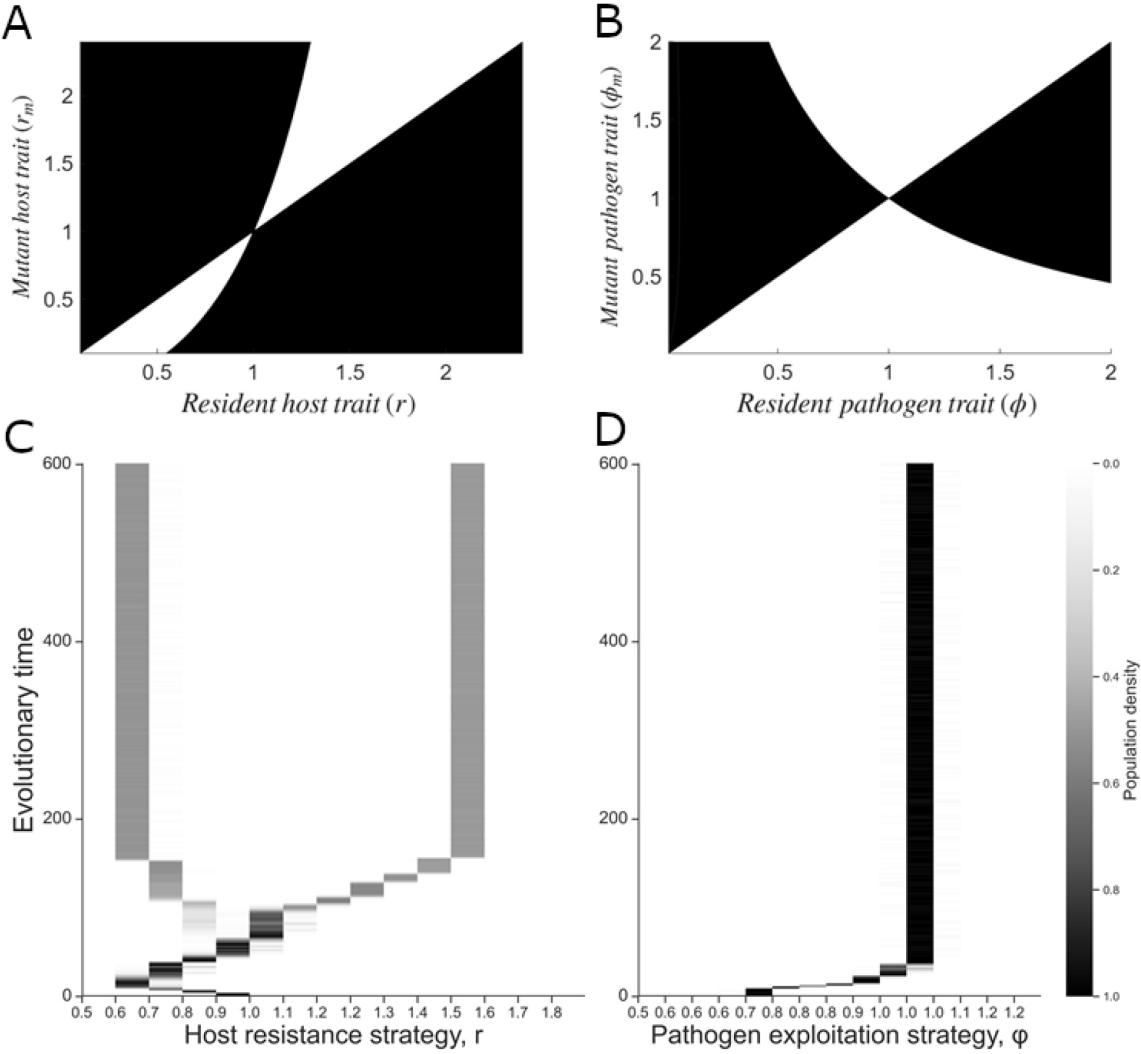
Coevolutionary outcomes. PIP plots showing the occurrence of (A) evolutionary branching point in the host resistance strategy *r*, and **(B)** CSS in the pathogen exploitation strategy *ϕ*. Shaded regions in (A) and (B) indicate the potential areas where mutant strains can invade the resident populations. **(C, D)** Output from numerical simulations confirming branching in the host strategy *r*, while the pathogen strategy *ϕ* stays at its CSS over evolutionary time. The parameters are set at a=2, τ=0.5, β=3, $\mu_{S}=0.2$, $\mu_{P}=0.2$, $\mu_{I}=0.2$, q=0.2, $c^{\prime }(r^{*})=0.3423$, $c^{\prime \prime }(r^{*})=-0.01$, $\mu^{\prime }(\phi^{*})=2.2$, and $\mu^{\prime \prime }(\phi^{*})=1$.

The pairwise invasibility plots (PIP) show the existence of a branching point in the host resistance strategy (Fig 5A), and a CSS in the pathogen exploitation strategy (Fig 5B). Plots comprise resident strategies on the x-axis and mutant strategies on y-axis and the shaded regions represent where mutant strategies can invade and replace the resident strategy. Singular points occur at the intersections of the fitness curve with the main diagonal. The evolutionary trajectory of the population proceeds incrementally along the diagonal via small mutation steps, either upward or downward, depending on selection pressures. In Fig 5A, the singular strategy is a branching point, meaning that it attracts nearby strategies but remains vulnerable to invasion by mutants both above and below the resident strategy. Over time, this results in disruptive selection, ultimately driving the coexistence of two distinct host strategies. On the other hand, Fig 5B shows a convergent stable strategy at 1, which attracts nearby strategies and resists invasion by any mutants, ensuring evolutionary stability.

We use numerical simulations to show the evolutionary trajectory demonstrating dimorphism in the host population while the pathogen remains monomorphic, following the algorithm outlined by [75]. As such, both populations initially evolve toward the singular point. However, when the dynamics approaches close to this point, the host population undergoes branching, resulting in two distinct subpopulations with different resistance traits (Fig 5C), while the pathogen population stabilizes at the singular point and remains monomorphic (Fig 5D). This outcome, however, is highly sensitive to the choice of our trade-off shapes. Here we consider 15 strains of each population and initialize the system with all densities set to zero, except for a single strain of each type. Then we numerically solve the population dynamics for a time sufficient for the populations to reach their equilibria. We run the simulation for T=600 mutation events. During each mutation event, a small density of a mutant strain is introduced into one of the populations– *S* (susceptible), *I* (infected), or *P* (free pathogens)– with equal probability. The mutant strains are generated by introducing small random deviations around the current trait values. After the addition of the mutant, we run a long-term simulation of epidemic dynamics. At the end of each simulation, any strain with a density below 0.005 is considered extinct and set to zero. This iterative process is repeated to track the directional evolution of both populations (see [13]).

However, evolutionary branching in the host population is highly sensitive to the choice of trade-off shape; branching occurs only under a narrow range of weakly accelerating trade-offs, where the cost of increasing resistance rises slowly at first and then more steeply. Under stronger accelerating or decelerating trade-offs, the population evolves towards either an evolutionarily stable strategy or a repeller strategy.

## Discussion

We consider a scenario where the pathogen exists both within hosts and as free-living propagules, and transmission can occur solely through environmental exposure to these propagules, as in the *Daphnia magna-Pasteuria ramosa* system and in amphibian chytridiomycosis caused by *Batrachochytrium dendrobatidis* [67,82]. Within this population-level framework, we examine coevolutionary dynamics shaped by two trade-offs: the cost of host resistance expressed via reduced host survival, and pathogen exploitation strategy that enhances propagule replication but shortens infected host lifespan.

Three key insights emerge from our model analysis: (i) stable investment in host resistance evolves to peak at intermediate levels of infection transmission and infected host mortality, forming a downward U-shaped pattern; (ii) coevolution of host and pathogen strategies changes patterns of free pathogen abundance, particularly in response to increasing infected and susceptible host mortality rates, as compared to when no strategies evolve; and (iii) dimorphic host strains with distinct levels of resistance can coexist through evolutionary branching at particular tradeoff configurations.

Models have examined how parasite exploitation strategies evolve by treating virulence as a function of both exploitation strategy and immune-mediated host mortality [48]. In Day’s framework, immune-mediated damage, which they refer to as immunopathology, is considered as general mortality cost of defense arising from either misdirected immune activation, energetic constraints, or resource reallocation. Their findings predicted that selection favors moderate exploitation strategies when immune-associated damage increases with parasite exploitation, and highly virulent parasites are selected when such damage is independent of parasite caused exploitation. Our study extends this line of work by shifting the focus from pathogen evolution to how host defense and pathogen exploitation coevolves, in an environmental transmission framework. Our key focus is to uncover how such survival-based costs of defense interact to shape the optimal investment in host resistance.

We find that host resistance becomes too costly to maintain when either hosts or free pathogen propagules are scarce at high mortality rates, and is therefore minimized under such conditions. Similarly, when infection transmission reaches extreme levels, selection favors reduced investments in host resistance, as either the cost of avoiding propagules becomes too high or such defense strategy becomes ineffective when infection is nearly unavoidable. This pattern of high transmission selecting for less resistance matches the findings of a within-host model (no free propagules) where the cost of resistance manifests as lower tolerance, i.e., higher mortality [80], and has also been observed in a system of rust fungi, *Puccinia punctiformis* and its host *Circium arvense* [83]. Furthermore, we discovered that free-pathogen density declines with increasing transmission rates, because rapid infection turnover leads to quicker host depletion, limiting opportunities for pathogen replication. This suggests that under certain trade-off conditions, increased invasion efficiency of pathogens within the host may paradoxically select against strong immune responses, thereby limiting both self-inflicted damage and pathogen load.

Within-host models of evolution that exclude a free-living stage of the parasite species often focus on a core question in host immune defense evolution: how defense strategies shape and are shaped by parasite prevalence in the host population [39,81]. Some studies have extended this framework to examine how evolutionary feedbacks shape infection prevalence compared to outcomes from purely ecological scenarios (without evolving strategies) [80,84], revealing that the evolution of host defense strategies and their associated costs can significantly alter pathogen prevalence patterns. In this study, we track how the population density of free propagules changes in response to different parameters at coevolutionary stable levels of host immunity and pathogen exploitation. By comparing these outcomes with an ecological scenario where no traits evolve, we discovered that pathogen density peaks at parameter ranges equivalent to intermediate levels of infection pressure. However, in evolving systems, host resistance and pathogen exploitation strategies continue to adapt, shifting pathogen density peaks toward conditions that sustain higher infection burdens. These results highlight how evolutionary pressures can shift infection dynamics, reinforcing the interplay between host adaptation, pathogen exploitation, and the costs of immune defense.

The coexistence of multiple host resistance genotypes within a population is a well-documented yet intriguing phenomenon, prompting questions about the conditions that sustain such diversity. Empirical studies have observed this pattern in both plant disease systems [85–89], and in animal diseases [47,90–93]. Several coevolutionary models have explored this phenomenon in specific disease systems [3,62,94,95], particularly within frameworks where transmission occurs directly between hosts [14–16,96]. As per our knowledge, only a few models have examined the possibilities of polymorphism in systems involving environmental transmission via free-living propagules [21,22,34]. [21] demonstrated that if the loss in direct pathogen transmission is overcompensated by the gain in environmental transmission, two distinct strains of pathogen population can emerge and coexist. Similarly, [22] showed that a combination of virulence-transmission, virulence-shedding, and virulence-persistence trade-offs can generate coexisting pathogen strains of different virulence levels. Our model, based on an environmentally transmitted pathogen system and incorporating immune-mediated costs, shows that diversification can arise in host resistance trait, leading to the coexistence of host strains with distinct resistance levels, as observed empirically in *Arabidopsis thaliana-Pseudomonas syringae* system [97,98]. However, such diversification occurs only when the costs of resistance increase slowly at higher investment levels, corresponding to a weakly accelerating cost function. Furthermore, we found no branching in the pathogen exploitation strategy, indicating that host resistance diversification does not necessarily drive diversification in pathogen traits.

To conclude, our theoretical framework extends classic models of host-pathogen coevolution to environmentally transmitted systems, capturing how the costs of host resistance and pathogen exploitation jointly shape evolutionary outcomes. A limitation is that parameter values were chosen to ensure infection persistence rather than derived from empirical data; future work could address this by using experimentally informed parameters or testing the robustness of outcomes across broader parameter ranges. The model could be further extended to include more ecological complexities–for instance, by incorporating direct host-to-host transmission to study how mixed transmission modes affect coevolution. Further developments may also include spatial structure, multiple pathogen strains, varying mutation rates, or defense mechanisms varying in specificity and cost. Environmentally transmitted pathogen systems, where host immune responses induces self-inflicted damage or lytic release of pathogens, offer tractable avenues for testing our theoretical findings. Empirical validation of our theoretical insights shall contribute to improved disease management and crop-protection strategies.

## Supporting information

S1 Text Detailed analysis of the host fitness calculation, basic reproduction number, and local stability of the endemic equilibrium. (PDF)

S1 Fig PDF consisting of the additional plots for CSS resistance variation and sensitivity analysis. (PDF)

S1 Data Mathematica notebook PDF demonstrating the occurrence of branching in the susceptible host population through signs of mutual invasibility condition. (PDF)

S2 Data Mathematica notebook PDF demonstrating numerical verification of the Routh-Hurwitz conditions for local stability of the endemic equilibrium. (PDF)
