# Supplementary material for: Coevolution of host resistance and pathogen exploitation in a propagule-mediated infection model
Source: PLoS Comput Biol. 2026 Mar 10;22(3):e1013999. doi: 10.1371/journal.pcbi.1013999 (PMC12998951; doi:10.1371/journal.pcbi.1013999)
Supplement: S1 Text — (PDF) [file pcbi.1013999.s001.pdf]

Supplementary Information: Coevolution of host resistance and pathogen exploitation in a propagule-mediated infection model

P. Singh, J. Sheen, C. Saad-Roy, M. Z. Levy, C. J. E. Metcalf

1 Model analysis

The population-dynamics governing the coevolution of host resistance and pathogen exploitation strategy structured around host immune-mediated damage and pathogen survival is given by the following system of equations:

$$\begin{aligned}\frac{dS}{dt} &= (a - q(S+I))S - (\beta - r)SP - \mu_S S - c(r)S,\\ \frac{dI}{dt} &= (\beta - r)SP - \mu_I I - \mu(\phi)I,\\ \frac{dP}{dt} &= \tau\phi I - \mu_P P.\end{aligned} \tag{1}$$

The endemic equilibrium is given by (S^*, I^*, P^*), where

$$\begin{aligned}S^* &= \frac{-(\mu(\phi)+\mu_I)\mu_P}{(r-\beta)\tau\phi},\\ I^* &= -\frac{\mu_P\left(q\left(\mu(\phi)+\mu_I\right)\mu_P + (r-\beta)\tau\phi(a - c(r) - \mu_S)\right)}{(r-\beta)\tau\phi\left((r-\beta)\tau\phi - q\mu_P\right)},\\ P^* &= \frac{-q\left(\mu(\phi)+\mu_I\right)\mu_P + (r-\beta)\tau\phi(-a + c(r) + \mu_S)}{(r-\beta)\left((r-\beta)\tau\phi - q\mu_P\right)}.\end{aligned}$$

The basic reproduction number is given by

$$\mathcal{R}_0 = \frac{(\beta - r)\, S^0\, \tau\phi}{(\mu(\phi) + \mu_I)\, \mu_P} = \frac{(\beta - r)\, \tau\phi\, (a - c(r) - \mu_S)}{(\mu(\phi) + \mu_I)\, \mu_P\, q}.$$

We prove the local stability of this equilibrium numerically in Mathematica software, using Routh Hurwitz stability conditions [4]. As such, we first derive the trace and determinant of the Jacobian matrix J of the above system of equations (check Mathematica notebook for Jacobian matrix). To satisfy the local stability conditions, we need

$$\begin{aligned} Tr(J) &< 0, \\ Det(J) &< 0, \\ \text{Sum of } 2{\times}2 \text{ principal minors of } J &> 0, and \\ \mathrm{Tr}(J) \cdot (\text{Sum of } 2{\times}2 \text{ minors}) - Det(J) &> 0. \end{aligned}$$

The trace is given by

$$Tr(J) = -\mu - \mu_I - \mu_P - \frac{3q(\mu + \mu_I)\mu_P}{(\beta - r)\, \tau\, \phi} + \frac{2q\, \mu_P (a + \mu + \mu_I - c - \mu_S)}{q\, \mu_P + (\beta - r)\, \tau\, \phi}.$$

The determinant is given by
$Det(J) = \mu_P \left(-(\mu + \mu_I)(a - c - \mu_S) - \frac{q(\mu+\mu_I)(4\mu-3\mu+\mu_I)\mu_P}{(r-\beta)\,\tau\,\phi} - \frac{2q(\mu-\mu)\mu_P(a-c+\mu+\mu_I-\mu_S)}{q\,\mu_P+(r-\beta)\,\tau\,\phi} \right)$. Given complicated mathematical expressions, we demonstrated the required stability conditions in Mathematica using parameter values.

1.1 Biological constraints and parameter set interpretation

As suggested in related target cell based within-host model, individual parameters are often best interpreted in combinations than in isolation, as biological constraints typically apply to composite quantities (for e.g burst size, see [1]). Similarly, in our propagule-mediated model, the total propagule output generated during a single infection duration is jointly determined by the parameters τ and ϕ.

In our model, infected hosts are removed at total mortality rate $\mu_I + \mu(\phi)$, so the mean infection duration is given by

$$T_i = \frac{1}{\mu_I + \mu(\phi)}.$$

As propagules are released at rate $\tau\phi$, the total propagule release per infection is given by

$$P_I = \tau\phi T_1 = \frac{\tau\phi}{\mu_I + \mu(\phi)}.$$

Furthermore, environmental persistence of free propagules for the mean time is

$$T_p = \frac{1}{\mu_P}.$$

Using the default values (Table 1), we get $T_i = 0.83$, $P_I = 0.83$, and $T_p = 5$. Besides,

$$\mathcal{R}_0 = \frac{(\beta - r)\, S^0\, \tau\phi}{(\mu(\phi) + \mu_I)\, \mu_P} = (\beta - r)\ S^0 P_I T_p = 33.3.$$

Thus, conditions for invasion and persistence are achieved not only by selecting $\mathcal{R}_0 > 1$, but by ensuring that the infection duration, propagule output per infection, and environmental persistence remain finite and feasible.

1.2 Host invasion fitness analysis

Next, we derive the invasion fitness expressions for the mutant host and pathogen strains, under the assumptions that higher investment in host resistance r incurs cost in terms of increased background host mortality rate $c(r)$, and pathogen exploitation strategy ϕ incurs cost via reduced pathogen survival within the host $\mu(\phi)$. For the host, the mutant strain with strategy $(r, c(r_m))$ tries to invade the resident strain with strategy (r, c) which is currently fixed at its stable equilibrium. To determine the possibility of invasion by this mutant host strain, we calculate its

invasion fitness using the mutant dynamics at low densities:

$$\begin{aligned}\frac{dS_m}{dt} &= (a - q(S^* + I^* + S_m + I_m))S_m - (\beta - r_m)S_m(P^* + P_m) - \mu_S S_m - c(r_m)S_m,\\ \frac{dI_m}{dt} &= (\beta - r_m)S_m(P^* + P_m) - \mu_I I_m - \mu(\phi_m)I_m.\end{aligned} \tag{2}$$

Following the second order approximation error i.e., $N_m^2 << N^2$, the Jacobian matrix is given by,

$$J_m = \begin{pmatrix} \frac{\partial S_m'}{\partial S_m} & \frac{\partial S_m'}{\partial I_m} \\ \frac{\partial I_m'}{\partial S_m} & \frac{\partial I_m'}{\partial I_m} \end{pmatrix} = \begin{pmatrix} a - q(S^*(r) + I^*(r)) - (\beta - r_m)P^*(r) - \mu_S - c(r_m) & 0 \\ (\beta - r_m)P^*(r) & -\mu_I - \mu(\phi_m) \end{pmatrix}.$$

$$= \begin{pmatrix} A & B \\ C & D \end{pmatrix}.$$

The eigenvalues of J_m are:

$$\lambda_+, \lambda_- = \frac{(A + D) \pm \sqrt{(A + D)^2 - 4(AD - BC)}}{2} = \frac{(A + D) \pm \sqrt{(A - D)^2 + 4BC}}{2}, \tag{3}$$

where the subscript on λ denote its sign taken in the solution. Through positivity of parameters and $(\beta - r_m) > 0$, it is clear that $C > 0$, $B = 0$, $D < 0$, and the sign of A remains unknown. As $\sqrt{(A - D)^2 + 4BC} > 0$, we always get two real eigenvalues. From the analysis of [3], we know that if $D < 0$ then the smaller eigenvalue i.e. λ_- is always negative. Then the sign of the determinant of J_m always depend upon the sign of larger eigenvalue λ_+. Finally, the host invasion fitness is equivalent to the maximum eigenvalue of this matrix [6], and is given by

$$s(r, r_m) = a - q(S^*(r) + I^*(r)) - (\beta - r_m)P^*(r) - \mu_S - c(r_m).$$

The host selection gradient is given by the derivative of the invasion fitness expression with respect to the mutant strategy r_m [2], as follows:

$$\frac{\partial s(r, r_m)}{\partial r_m} = P^*(r) - c'(r_m). \tag{4}$$

The analysis for fitness expression of the mutant pathogen strain with strategy ϕ_m which is introduced into the resident environment with strategy ϕ is given in the main text.

1.3 Stability conditions for co-CSS

The evolutionary behaviour at a cosingular strategy depends upon the sign of second-order derivatives of fitness gradients of both species. Similar to single-species evolution case, for respective host and pathogen strategy to be evolutionary stable (ES) in coevolutionary framework, we need

$$EH = \frac{\partial^2 s}{\partial r_m^2} < 0, \tag{5}$$

$$EP = \frac{\partial^2 p}{\partial \phi_m^2} < 0. \tag{6}$$

The cosingularity (r^*, ϕ^*) will be evolutionary stable when $EH < 0$ and $EP < 0$. Convergence stability, on the other hand, is verified by the Jacobian matrix formed of the second order derivatives of the host and pathogen fitness expressions,

$$J = \begin{pmatrix} \eta_h X^* \big(\frac{\partial^2 s}{\partial r_m^2} + \frac{\partial^2 s}{\partial r \partial r_m}\big) & \eta_h X^* \big(\frac{\partial^2 s}{\partial r_m \partial \phi}\big) \\ \eta_p Y^* \big(\frac{\partial^2 p}{\partial \phi_m \partial r}\big) & \eta_p Y^* \big(\frac{\partial^2 p}{\partial \phi_m^2} + \frac{\partial^2 p}{\partial \phi_m \partial \phi}\big) \end{pmatrix} = \begin{pmatrix} \eta_h X^*(EH + MH) & \eta_h X^* A_H \\ \eta_p Y^* A_P & \eta_p Y^*(EP + MP) \end{pmatrix},$$

evaluated at the co-singularity [5]. From the Routh-Hurwitz criteria, the cosingularity is convergence stable if and only if determinant $J > 0$ and Trace $J < 0$. Following conditions are

sufficient to satisfy this criterion:

$$EH + MH < 0, \tag{7}$$

$$EP + MP < 0, \tag{8}$$

$$(EH + MH)(EP + MP) > |A_H A_P| \tag{9}$$

Conditions 7 and 8 are equivalent to the condition of convergence stability in a single-species evolution scenario. Condition 9 is a sufficient but not necessary to confirm a co-CSS.

References

[1] Katharine Best and Alan S Perelson. Mathematical modeling of within-host zika virus dynamics. *Immunological reviews*, 285(1):81–96, 2018.

[2] Stefan AH Geritz, Géza Mesze, Johan AJ Metz, et al. Evolutionarily singular strategies and the adaptive growth and branching of the evolutionary tree. *Evolutionary ecology*, 12(1): 35–57, 1998.

[3] Andrew Hoyle, Alex Best, and Roger Bowers. Evolution of host resistance towards pathogen exclusion: the role of predators. *Evol. Ecol. Res.*, 14(2):125–146, 2012.

[4] Amy Hurford, Daniel Cownden, and Troy Day. Next-generation tools for evolutionary invasion analyses. *Journal of the Royal Society Interface*, 7(45):561–571, 2010.

[5] Éva Kisdi. Trade-off geometries and the adaptive dynamics of two co-evolving species. *Evol. Ecol. Res*, 8(6):959–973, 2006.

[6] JAJ Metz, SAH Geritz, and G Meszéna. Adaptive dynamics, a geometrical study of the consequences of nearly faithful reproduction. *Int. Inst. Appl. Syst. Anal.*, page 42, 1995.
