# Supplementary material for: Coevolution of host resistance and pathogen exploitation in a propagule-mediated infection model
Source: PLoS Comput Biol. 2026 Mar 10;22(3):e1013999. doi: 10.1371/journal.pcbi.1013999 (PMC12998951; doi:10.1371/journal.pcbi.1013999)
Supplement: S1 Fig — (PDF) [file pcbi.1013999.s002.pdf]

# Supplementary Information: Additional figures

P. Singh, J. Sheen, C. Saad-Roy, M. Z. Levy, C. J. E. Metcalf

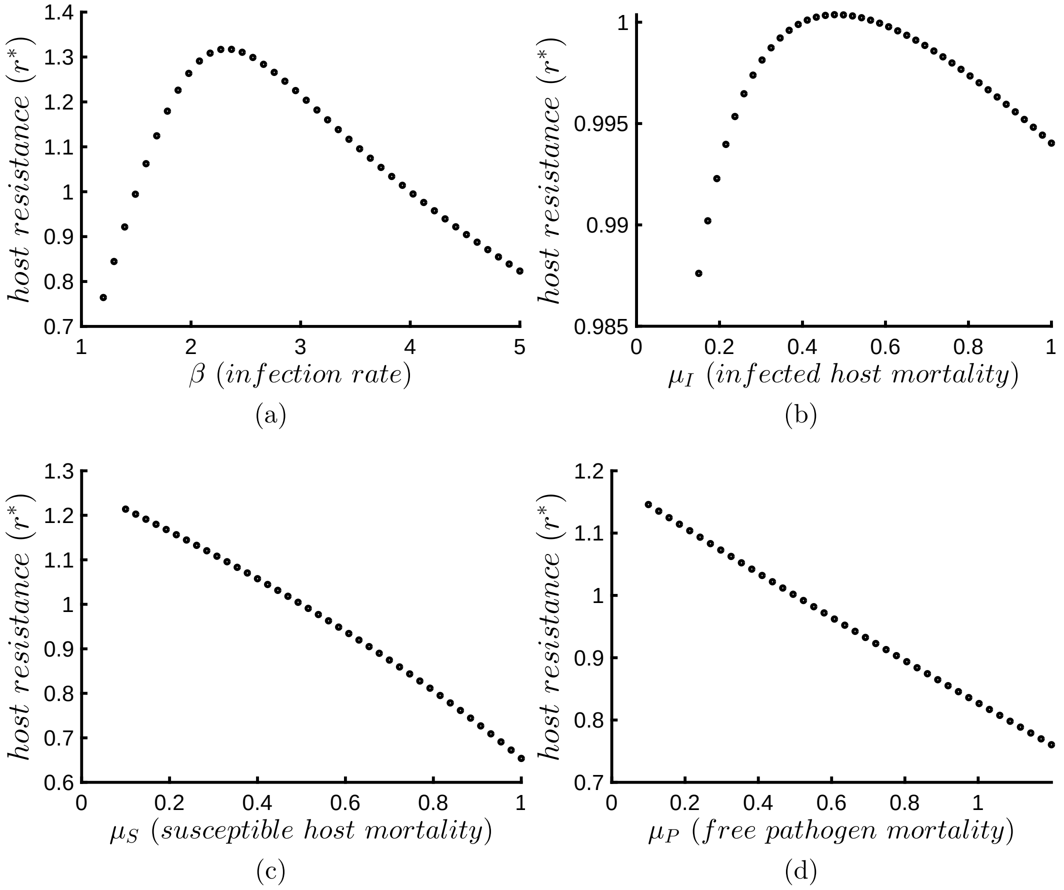


Figure 1: *Additional plots showing how CSS investment in resistance varies with increasing* $\beta$*,* $\mu_I$*,* $\mu_S$*, and* $\mu_P$*, for a different parameter set. Except for the varying parameter in each figure, the default parameter set used here is* $a = 3$*,* $q = 0.1$*,* $\beta = 4$*,* $\mu_I = 0.4$*,* $\mu_S = 0.5$*,* $\mu_P = 0.5$*,* $c'(r^*) = 0.4$*,* $\mu'(\phi^*) = 2.4$*,* $c''(r^*) = 0.5$*, and* $\mu''(\phi^*) = 1$*.*

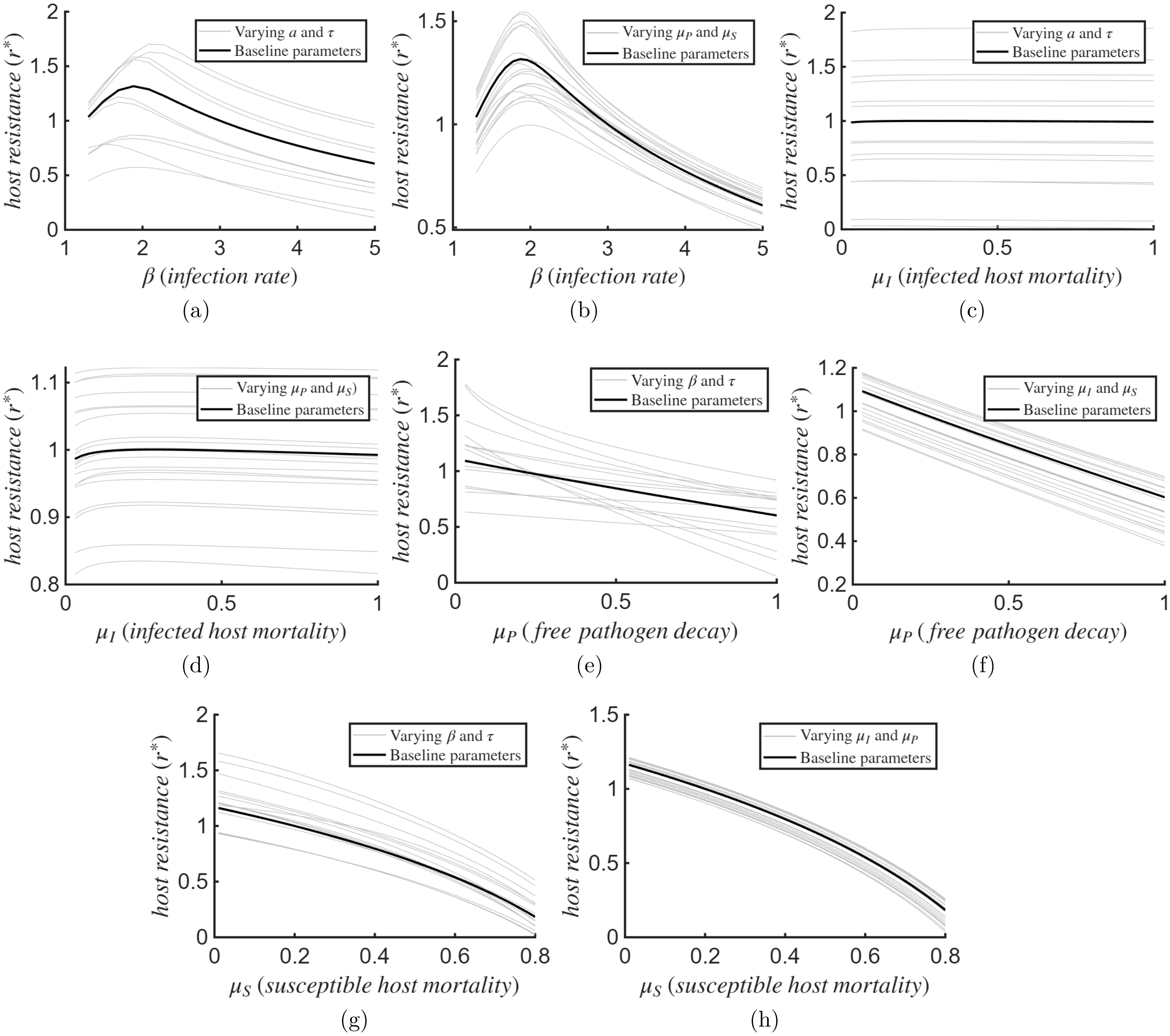


Figure 2: *Additional plots showing the robustness of CSS patterns across broad range of nuisance parameters while retaining only biologically feasible endemic equilibria* $(S^*, I^*, P^*) > 0$. *Dark grey lines show the CSS pattern obtained under the default parameter set, whereas thin grey lines represent CSS patterns obtained by varying nuisance parameters (including* $a$, $\tau$, $\mu_S$, $\mu_P$, $\mu_I$ *and* $\beta$*) within predefined ranges. The persistence of the qualitative trends demonstrates that the patterns reported in Fig. 3 in the main text are valid across a wide region of feasible parameter space.*
