# Supplementary material for: Coevolution of host resistance and pathogen exploitation in a propagule-mediated infection model
Source: PLoS Comput Biol. 2026 Mar 10;22(3):e1013999. doi: 10.1371/journal.pcbi.1013999 (PMC12998951; doi:10.1371/journal.pcbi.1013999)
Supplement: S1 Data — (PDF) [file pcbi.1013999.s003.pdf]

In[108]:=

```
Clear["Global`*"];

RealQ[x_] := Element[x, Reals] === True;

dX[X_, Y_, P_] := (a - q (X + Y)) X - (β - r) X P - mus X - c X;
dY[X_, Y_, P_] := (β - r) X P - mui Y - mu Y;
dP[X_, Y_, P_] := τ ϕ Y - mup P


(*Sol=Solve[{dX[X,Y,P]==0,dY[X,Y,P]==0, dP[X,Y,P]==0},{X,Y,P}]*)
c[r_] := (1 - r1^2 (1 - Exp[r2 (r - 1) / r1]) / r2);
mu[ϕ_] := (2 - m1^2 (1 - Exp[m2 (ϕ - 1) / m1]) / m2);

X1[r_, ϕ_] := - ((mu[ϕ] + mui) mup) / ((r - β) τ ϕ);
Y1[r_, ϕ_] := - (mup (q (mu[ϕ] + mui) mup + (r - β) τ ϕ (a - c[r] - mus))) / ((r - β) τ ϕ ((r - β) τ ϕ - q mup));
P1[r_, ϕ_] := (-q (mu[ϕ] + mui) mup + (r - β) τ ϕ (-a + c[r] + mus)) / ((r - β) ((r - β) τ ϕ - q mup));
(*Sstar=-((mu+μI)*μp)/((r-β)*τ*ϕ);
Istar=-(μp*(q*(mu+μI)*μp+(r-β)*τ*ϕ*(a-c-μS)))/((r-β)*τ*ϕ*((r-β)*τ*ϕ-q*μp));
Pstar=(-q*(mu+μI)*μp+(r-β)*τ*ϕ*(-a+c+μS))/((r-β)*((r-β)*τ*ϕ-q*μp));*)


(*gradhost = Pstar
   gradpar = (μI+mu)/ϕ*)
```

In[118]:=

```
r1 = 0.3423; r2 = -0.01; m1 = 2.2; m2 = 1;
s[r_, rm_, ϕ_] := a - q (X1[r, ϕ] + Y1[r, ϕ]) - (β - rm) P1[r, ϕ] - mus - c[rm];
p1[ϕ_, ϕm_, r_] := (ϕm (mui + mu[ϕ])) / (ϕ (mui + mu[ϕm])) - 1;
```

In[121]:=

```
hostgrad[r_, ϕ_] := (Evaluate[D[s[r, rm, ϕ], rm]]) /. {rm → r}; (*host gradient*)
pargrad[r_, ϕ_] := (Evaluate[D[p1[ϕ, ϕm, r], ϕm]]) /. {ϕm → ϕ};
(*parasite gradient*)

EH[r_, ϕ_] := (Evaluate[D[s[r, rm, ϕ], {rm, 2}]]) /. {rm → r}; (*host ESS*)
EP[r_, ϕ_] := (Evaluate[D[p1[ϕ, ϕm, r], {ϕm, 2}]]) /. {ϕm → ϕ};  (*pARASITE ESS*)

dsdrm[r_, rm_, ϕ_] := D[s[r, rm, ϕ], rm];
MH[r_, ϕ_] := Evaluate[(Evaluate[D[dsdrm[r, rm, ϕ], r]]) /. {rm → r}];
(*host MI*)

drdγm[ϕ_, ϕm_, r_] := D[p1[ϕ, ϕm, r], ϕm];
MP[r_, ϕ_] := Evaluate[(Evaluate[D[drdγm[ϕ, ϕm, r], ϕ]]) /. {ϕm → ϕ}];
```

In[129]:=

```
a = 2; β = 3; τ = 0.5; mus = 0.2;
mup = 0.2; mui = 0.2; q = 0.2; ϕ = 1;
mu = 2;
EH[1, 1]
MH[1, 1]
EH[1, 1] + MH[1, 1]
MP[1, 1]
```

Out[130]=

```
0.01
```

Out[131]=

```
-0.0211501
```

Out[132]=

```
-0.0111501
```

Out[133]=

```
0.
```
