# Supplementary material for: Coevolution of host resistance and pathogen exploitation in a propagule-mediated infection model
Source: PLoS Comput Biol. 2026 Mar 10;22(3):e1013999. doi: 10.1371/journal.pcbi.1013999 (PMC12998951; doi:10.1371/journal.pcbi.1013999)
Supplement: S2 Data — (PDF) [file pcbi.1013999.s004.pdf]

In[183]:=

```
(*Clear all previous definitions*)
ClearAll[S, Ip, P, a, q, β, r, μS, c, μI, mu, τ, ϕ, μp]

(*Define variables and parameters*)
vars = {S, Ip, P};
params = {a, q, β, r, μS, c, μI, mu, τ, ϕ, μp};

(*Define the system of ODEs*)
f1 = (a - q * (S + Ip)) * S - (β - r) * S * P - μS * S - c * S;
f2 = (β - r) * S * P - μI * Ip - mu * Ip;
f3 = τ * ϕ * Ip - μp * P;

(*Compute the Jacobian matrix*)
J = D[{f1, f2, f3}, {{S, Ip, P}}];

(*Define endemic equilibrium values*)
Sstar = - ((mu + μI) * μp) / ((r - β) * τ * ϕ);
Istar = - ((μp * (q * (mu + μI) * μp + (r - β) * τ * ϕ * (a - c - μS))) /
      ((r - β) * τ * ϕ * ((r - β) * τ * ϕ - q * μp)));
Pstar = (-q * (mu + μI) * μp + (r - β) * τ * ϕ * (-a + c + μS)) / ((r - β) * ((r - β) * τ * ϕ - q * μp));

(*Substitute the equilibrium into the Jacobian*)
Jstar = J /. {S → Sstar, Ip → Istar, P → Pstar};

MatrixForm[Jstar]
```

Out[194]//MatrixForm=

$$\left(\begin{array}{l} a - c - \mu S + \frac{q\ (mu+\mu I)\ \mu p}{(r-\beta)\ \tau\ \phi} - \frac{(-r+\beta)\ (-q\ (mu+\mu I)\ \mu p+(r-\beta)\ (-a+c+\mu S)\ \tau\ \phi)}{(r-\beta)\ (-q\ \mu p+(r-\beta)\ \tau\ \phi)} - q \left(- \frac{(mu+\mu I)\ \mu p}{(r-\beta)\ \tau\ \phi} - \frac{\mu p\ (q\ (mu+\mu I)\ \mu p+(r-\beta)\ (a-c-\mu S}{(r-\beta)\ \tau\ \phi\ (-q\ \mu p+(r-\beta)\ \tau\ \phi)} \right. \\ \frac{(-r+\beta)\ (-q\ (mu+\mu I)\ \mu p+(r-\beta)\ (-a+c+\mu S)\ \tau\ \phi)}{(r-\beta)\ (-q\ \mu p+(r-\beta)\ \tau\ \phi)} \\ 0 \end{array}\right.$$

In[195]:=

```
TraceJ := -mu - μI - μp + (3 q (mu + μI) μp)/((r - β) τ ϕ) + (2 q μp (a - c + mu + μI - μS))/(q μp + (-r + β) τ ϕ);

DeterJ = μp (-((mu + μI) (a - c - μS)) -
     (q (mu + μI) (4 mu - 3 mu + μI) μp)/((r - β) τ ϕ) - (2 q (mu - mu) μp (a - c + mu + μI - μS))/(q μp + (-r + β) τ ϕ));

R := (τ ϕ (β - r) (a - c - μS))/(q μp (μI + mu));

principalMinors2x2[Jstar_] :=
 Module[{indices, minors}, indices = Subsets[Range[Length[Jstar]], {2}];
  minors = Det[Jstar[[#, #]]] & /@ indices;
  minors]

SumOfPrincipalMinors = Total[principalMinors2x2[Jstar]];
```

In[200]:=

```
RHCondition = TraceJ * SumOfPrincipalMinors - DeterJ;
third = (((-mu - μI) (-μp) + ((-r + β) (mu + μI) μp)/((r - β) τ ϕ) (τ ϕ)) +
    ((a - c - μS + (q (mu + μI) μp)/((r - β) τ ϕ) - ((-r + β) (-q (mu + μI) μp + (r - β) (-a + c + μS) τ ϕ))/((r - β) (-q μp + (r - β) τ ϕ)) -
        q (-((mu + μI) μp)/((r - β) τ ϕ) + (μp (q (mu + μI) μp + (r - β) (a - c - μS) τ ϕ))/((r - β) τ ϕ (-q μp + (r - β) τ ϕ))))
      (-μp) - (((-r + β) (mu + μI) μp)/((r - β) τ ϕ)) (0)) +
    ((a - c - μS + (q (mu + μI) μp)/((r - β) τ ϕ) - ((-r + β) (-q (mu + μI) μp + (r - β) (-a + c + μS) τ ϕ))/((r - β) (-q μp + (r - β) τ ϕ)) -
        q (-((mu + μI) μp)/((r - β) τ ϕ) + (μp (q (mu + μI) μp + (r - β) (a - c - μS) τ ϕ))/((r - β) τ ϕ (-q μp + (r - β) τ ϕ)))) (-mu - μI) -
      ((q (mu + μI) μp)/((r - β) τ ϕ)) (((-r + β) (-q (mu + μI) μp + (r - β) (-a + c + μS) τ ϕ))/((r - β) (-q μp + (r - β) τ ϕ)))));
```

```
a = 2; β = 3; τ = 0.5; μS = 0.2; μp = 0.2; μI = 0.2; q = 0.2; r = 1; ϕ = 1; c = 1 ; mu = 2;
```

In[203]:=

```
TraceJ
DeterJ
SumOfPrincipalMinors
TraceJ SumOfPrincipalMinors - DeterJ
```

Out[203]=

```
-2.31785
```

Out[204]=

```
-0.97328
```

Out[205]=

```
0.398369
```

Out[206]=

```
0.0499214
```
